# Phenolic, Polysaccharides Composition, and Texture Properties during Ripening and Storage Time of New Table Grape Cultivars in Chile

**DOI:** 10.3390/plants12132488

**Published:** 2023-06-29

**Authors:** Alvaro Peña-Neira, Mariona Gil i Cortiella, Cristina Ubeda, Claudio Pastenes, Luís Villalobos, Loreto Contador, Rodrigo Infante, Camila Gómez

**Affiliations:** 1 Department of Agro-Industry and Enology, Facultad de Ciencias Agronómicas, Universidad de Chile, Santa Rosa 11315, La Pintana, Santiago 8820000, Chile; 2 Instituto de Ciencias Químicas Aplicadas, Facultad de Ingeniería, Universidad Autónoma de Chile, Av. El Llano Subercaseaux 2801, San Miguel, Santiago 8910060, Chile; mariona.gil@uautonoma.cl; 3 Área de Nutrición y Bromatología, Departamento de Nutrición y Bromatología, Toxicología y Medicina Legal, Facultad de Farmacia, Universidad de Sevilla, C/P. García González no. 2, E-41012 Sevilla, Spain; c_ubeda@us.es; 4 Department of Plant Production, Facultad de Ciencias Agronómicas, Universidad de Chile, Santa Rosa 11315, La Pintana, Santiago 8820000, Chile; cpastene@uchile.cl (C.P.); luisvillalobosg1@gmail.com (L.V.);

**Keywords:** *Vitis vinifera*, Crimson Seedless, Timco™, Krissy™, polyphenolic profile, gene expression, phenylpropanoid biosynthesis

## Abstract

The aim of this study is to determine the phenolic and polysaccharidic composition, texture properties, and gene expression of new seedless table grape cultivars Timco™ and Krissy™ and compare them to the traditional table grape variety Crimson Seedless (*Vitis vinifera* L.), during ripening and in commercial postharvest conditions. According to the results, phenolic compounds were present in very different proportions. The total anthocyanins responsible for skin color increased during maturation and the majority anthocyanin in the three cultivars was peonidin-3-glucoside, followed by malvidin-3-glucoside. The phenolic compounds presented a different behavior (decreasing or increasing) during postharvest. The total skin soluble polysaccharides decreased during ripening and postharvest in Crimson Seedless and Krissy™ and remained constant from technological maturity to postharvest storage in Timco™. In all cultivars, the majority soluble polysaccharide fraction was that with a molecular mass between 500 and 35 KDa. The skin mechanical properties of table grapes were good parameters for differentiating varieties, with better results for the new cultivars, compared to the traditional Crimson Seedless, especially in postharvest. Genes involved in the flavonoid pathway and cell wall metabolism in skins exhibited an increase in expression from veraison to remaining constant at the end of the berry ripening.

## 1. Introduction

Grape ripening has been studied for many years and harvesting fruit at optimal ripeness is essential for marketing and storing table grapes [1].

The quality of the grape berry at harvest is determined by several significant physiological and biochemical changes that occur simultaneously in the grape berry during the ripening phase.

Veraison, the first symptom of ripening, marks the start of major metabolic changes, including sugar accumulation, softening of the berries, anthocyanin formation, organic acid metabolism, and accumulation of flavor compounds [2,3,4].

In general, table grapes have bigger berries and firmer pulp compared to wine grapes. Due to their reduced propensity for withering and crushing, these characteristics make table grapes less vulnerable to harm during transportation. On the other hand, consumers generally prefer seedless varieties with medium-sized berries, crisp, thin skin, and a sweet taste [5,6]. At technological maturity, the quality of table grapes is determined by their appearance, physical, and chemical characteristics. While the balance between sweetness and acidity is a fundamental concept in assessing the quality of many fruits, such as table grapes [6], texture is an important factor in determining the quality of table grapes. The viticulture and postharvest industries may be interested in instrument mechanical properties to identify the potential of each variety and help meet market demands [7]. These mechanistic variables are associated with certain organoleptic properties and thus indirectly affect consumer acceptance of the product [7,8]. Grape berries’ loss of firmness during ripening has frequently been linked to the breakdown of cell walls, particularly pectic polysaccharides [9]. Changes in the structure and composition of the cell wall are the result of hydrolytic enzymes produced by the fruit, namely polygalacturonase (PG), pectinesterase (PE), *β*-galactosidase (*β*-GAL), pectate lyase (PL), and cellulase, resulting from complex interactions [10]. The activity of these enzymes and the expression of the genes encoding them differ between table grape varieties, which may explain differences in the firmness of the varieties at the time of harvest [9,10].

Furthermore, the appearance of grapes has a significant impact on their commercial value, and poorly colored red–pink varieties lead to low consumer acceptance [11]. There is a strong correlation between anthocyanin concentration and the grape skin color index [12].

Only during veraison do anthocyanins begin to form in the skin of red grapes. Many of the genes involved in the flavonoid pathway exhibit a dramatic increase in expression in skin cells at veraison, according to analysis of their patterns of expression. Despite variations in gene expression levels between grape varieties, expression of the gene encoding a glycosyl transferase involved in the last stages of anthocyanin production was positively linked with anthocyanin synthesis [3]. In addition to anthocyanins, grapes are rich in other phenolic compounds thought to have antioxidant properties and health benefits [5]. For this reason, table grapes can be considered as a product with functional properties, as they are rich in nutrients and antioxidants and thus have many health benefits [13].

About 80% of Chile’s production of table grapes is exported. Chile is the world’s and the Southern Hemisphere’s top exporter of table grapes, according to the United States Department of Agriculture (USDA). Grape exports for the 2019/2020 season reached approximately 657,000 tons [14,15]. In recent years, Chile has been introducing several new varieties from various national and international breeding programs to the world market, which are replacing traditional grape varieties such as Red Globe, Crimson Seedless, and Thompson Seedless. Chile is searching for grapes with a superior size, better condition, and taste [14,16]. Last season (2021/2022), traditional varieties (Red Globe, Crimson Seedless, Thompson, Flame, Sugraone, Autumn Royal) accounted for 48% of production, with the remaining 52% being over 30 licensed new varieties, such as Timco™, Sweet Celebration™, Arra15™, Allison™, Magenta™, Scarlotta™, Pristine™, Sable™, Krissy™, and Maylen.

Due to the lack of characterization of new varieties during ripening and postharvest storage, the aim of this study is to determine the phenolic and polysaccharidic composition, texture properties, and gene expression of new seedless table grape cultivars Timco™ and Krissy™ and compare them to the traditional table grape variety Crimson Seedless (*Vitis vinifera* L.), during ripening and in commercial postharvest conditions. We evaluated a variety of parameters including: (i) grape maturity indicators, (ii) phenolic composition and antioxidant activity of skins, (iii) skin soluble polysaccharides according to molecular mass distribution, (vi) texture properties, and (v) gene expression of transcriptional regulators and biosynthetic enzymes of the anthocyanin pathway and cell wall metabolism of berry skins, related to the synthesis of anthocyanins and color, as well as the texture of berries, respectively.

## 2. Results and Discussion

### 2.1. Evolution of Basic Physical and Chemical Variables

The berry weight, equatorial diameter, length, and technological maturity parameters such as total soluble solids (°Brix), TA (expressed as g H_2_SO_4_ equivalents per liter of juice), and pH of varieties investigated are shown in Table 1.

Berry size is the most important quality factor in the international table grape market. Preference for grape berries is affected by berry size, texture, skin thickness, and astringency. In seedless cultivars, berry size is usually increased by external application of gibberellin or cytokinins in the early stages of fruit development [17] as a routine agronomic practice in commercial table grape vineyards.

In general, berry weight and equatorial diameter increased to varying degrees during ripening for all studied table grapes. At commercial maturity (D4), the berries weighed between 294 and 522 mg, which corresponded to the Crimson Seedless and Timco™ varieties, respectively, and their weight decreased during storage, except for the Krissy™ cultivar. The greater standard deviation presented in the weight by Krissy™ during postharvest storage compared to the other varieties under study would indicate a greater heterogeneity in the berries, which could explain the slight increase observed toward the last sampling, considering that in each sampling during storage, berries are obtained from different bunches stored inside boxes. The length of the berries for all varieties showed a slight increase during ripening, unlike the equatorial diameter that increased in the same period with values in D4 in a range between 24.72 (Crimson Seedless) and 30.53 mm (Timco™). Both parameters remained unchanged during the postharvest period.

Sugar and organic acids are important to berry flavor. The total soluble solids (TSS) content is commonly used to assess the quality of table grapes and determine harvest ripeness. The TSS varied among cultivars at different sampling dates because of their early maturity and different ripening behavior. The TSS values for the three cultivars at technological maturity (D4) ranged from 18.73 (Crimson Seedless and Timco™) to 19.40 (Krissy™). This may be due to the phenomenon of evaporation during long-term storage [18]. Furthermore, as expected, all varieties showed a significant increase in the pH and a significant decrease in total acidity during ripening. During storage, the pH remained almost constant, while total acidity increased slightly in all three varieties. Our results concerning the physiochemical parameters agree with previous studies concerning table grapes’ ripening and postharvest storage [9,16,17].

### 2.2. Evolution of Phenolic Compounds and Antioxidants in Skins

Phenolics are good antioxidants because of their vulnerability to oxidation owing to their hydroxyl groups and unsaturated double bonds (18). The polyphenolic profile of grapes is affected by the variety, geographic, climatic, and agronomical conditions, among other factors [17,19,20,21].

The behavior in the evolution of total phenols during ripening was quite similar for the three cultivars. The total phenols values in skins at technological maturity (D4) ranged from 0.75 gallic acid mg/g FW (Timco™) to 1.51 gallic acid mg/g FW (Crimson Seedless). The values are like those reported for red table grapes by other authors [20,21].

During the postharvest period, it was possible to observe a decrease in total phenols for the Crimson Seedless samples. On the other hand, both Timco™ and Krissy™ showed an increase in the value of total phenols. Sheng et al. [22], for the Summer black variety, observed an increase in the total phenol content during 28 days of storage. These authors point out that this increase is greater with the exposure of the berries to UV radiation given an increase in the expression of the genes in the phenylpropanoid pathway that are still active postharvest.

All the varieties studied showed an increase in the content of total anthocyanins during maturation and up to the date of technological maturity (D4), the date on which the samples presented a range between 0.38 for Timco™ and 0.75 for Crimson Seedless (Table 2). This coincides with other authors who have observed an increase in the concentration of total anthocyanins from veraison to technological maturity [3,10,11]. Comparing both postharvest storage dates (54 and 75 days), the Crimson Seedless and Krissy™ samples showed a decrease in the total anthocyanin content, while the Timco™ ones did not experience changes.

According to Xie et al. [23], the concentration of anthocyanins in grape berries in advanced ripening processes is a balance between synthesis and degradation. These authors observed that although anthocyanin synthesis genes were highly expressed in the Yan73 grape cultivar at the late ripening stage, anthocyanins were markedly degraded, presumably by the action of enzymes peroxidase and polyphenol oxidase (PPO). This balance could favor degradation over time, especially in a long postharvest storage. This could explain the rapid decrease in anthocyanin content observed in the Timco™ samples from D4 (technological maturity) to D5, which subsequently maintained their concentration until D6. On the other hand, during postharvest storage, an early increase in anthocyanin content was observed in the cultivar of the table grape ‘Yaghouti’ (*Vitis vinifera* L.), an increase that would be associated with the synthesis of anthocyanins related to the increase in sugar concentration (TSS) during the first period of storage. After this period of increase, a subsequent decrease is also observed [24] which coincides with the behavior presented in postharvest storage by the cultivars Crimson Seedless and Krissy™, for TSS and total anthocyanins (Table 1 and Table 2).

Total tannins decreased in all varieties during ripening (Table 2). At the date of commercial harvest, the highest values were presented by Crimson Seedless (5.47) and Krissy™ (6.57) and the lowest by Timco™ (3.48). The values of the total tannins from skins at the time of technological maturity are within the values observed for 36 grape cultivars (*Vitis vinifera* L.) by other authors [25]. The evolution of tannins from veraison to technological maturity has been studied by several authors [26]. Some of them point out that most of the tannin synthesis occurs immediately after fruit set and ends several weeks before veraison, while the second stage of tannin accumulation occurs just before this point, where the maximum tannin levels are found [26,27]. After veraison, Bogs et al. [28] found that the expressed genes most relevant to tannin synthesis were no longer detectable. This may partly explain why tannins do not accumulate during ripening and the generally decreasing levels that we observed.

During postharvest, no significant changes were observed in any of the varieties studied. In contrast, Sheng et al. [22] observed that during the storage of table grapes of the Summer black variety, a rapid decrease in 21 days was appreciated for total tannins, and a steady decrease thereafter.

The antioxidant activity of grapes depends on the quantitative differences in phenolic compounds and the content of various other antioxidants, such as carotenoids and vitamin C, which decrease during ripening [29,30]. All grape varieties studied showed elevated ORAC values at technological maturity ranging from 2453 μmol TE/100 g (TimcoTM) to 4627 μmol TE/100 g (Crimson Seedless). In all the varieties, it was observed that the antioxidant capacity presented an early increase in the first stage of maturation and remained without significant differences until the commercial harvest. These results differ from those observed by other studies in that the antioxidant capacity decreased during the ripening of the berries [29,31,32]. This could be because these studies were carried out with grapes destined to produce wine that reach a higher state of maturation compared with table grapes and therefore there is a greater probability of a decrease in the compounds responsible for the antioxidant capacity. 

At the end of storage, the antioxidant activity levels in all the varieties (ranging from 2894–4450 μmol TE/100 g) did not lead to an excessive reduction in the antioxidant capacity of grapes. These results agree with Nicolosi et al. [33], who studied the evolution of the antioxidant capacity in postharvest storage for the table grape varieties Vittoria, Superior Seedless^®^, Italia, Crimson Seedless, Red Globe, and Black Pearl.

Determining the anthocyanin profile of grapes at different stages of maturity is important for understanding the phenolic changes that occur during grape berry development [34]. Anthocyanins monomers, delphinidin-3-glucoside, cyanidin-3-glucoside, petunidin-3-glucoside, peonidin-3-glucoside, and malvidin-3-glucoside were identified in table grapes of the varieties studied, except for Timco™ in which the presence of delphinidin-3-glucoside and petunidin-3-glucoside were not detected. In all varieties at harvest time (D4), the majority anthocyanin was peonidin-3-glucoside, followed by malvidin-3-glucoside, in agreement with other authors [33]. In this study, the content for the two main anthocyanins presented in Crimson Seedless, Timco™, and Krissy™ significantly increased toward the last weeks of ripening. 

The goal of postharvest storage techniques is to manipulate the metabolism of fruits during storage to extend the shelf life of produce. Postharvest treatments such as low temperatures, high CO_2_ concentrations, and controlled and modified atmosphere packaging slow down many metabolic processes, leading to natural deterioration and loss of quality [19]. However, some of these treatments adversely affect anthocyanin levels, which adversely affect fruit color and nutritional value. Postharvest cold storage is known to activate modulation in a variety of fruits, including table grapes, but may affect anthocyanin biosynthesis, degradation, or both [35]. During postharvest storage, a decrease in the concentration of the two major anthocyanins was observed for all varieties studied, when comparing the last sampling date (D6) with the technological maturity date (D4). This coincides with other authors [29], who observed during 60 days of postharvest storage (0–1 °C) the decrease in the anthocyanins delphinidin-3-glucoside, cyanidin-3-glucoside, pelargonidin-3-glucoside, and malvidin-3-glucoside in berries of the table grape cultivar Rishbaba.

### 2.3. Evolution of Skin Soluble Polysaccharides

The plant cell wall is a complex interconnected structure composed of polysaccharides, cell wall proteins, and polyphenols. During fruit ripening and postharvest storage, the chemical composition of cell walls and tissue structures changes, affecting the sensory, chemical, and physical properties of grapes [36,37].

Table 3 shows the results of soluble polysaccharides of the skins of the three varieties under study, during ripening and in postharvest storage. These pectin polysaccharides are the building blocks of pectin, which are released from the complex cell wall network by the actions of various types of endogenous and exogenous enzymes during ripening [37]. During ripening, the berry undergoes many compositional changes that affect the final chemical composition and overall polysaccharide profile at harvest and affect the firmness during and throughout the ripening period of the berry [38,39].

Large changes in specific polysaccharide components and in protein content are observed during softening and ripening [40]. Fasori et al. [41] reported that the most prominent changes in the grape skins from the ripening stage to mid-ripening to full ripeness were due to the modification of hemicellulose and pectin in the inner layer, mainly the modification of cellulose (pectin was more less) in the epidermal layer.

Pectin depolymerization and solubilization correlates with skin wall swelling, leading to the conclusion that pectin depolymerization and de-esterification are key processes leading to increased cell wall porosity and the softening of fruit during ripening.

In all varieties, a decrease in total polysaccharides was observed during ripening (Table 3), which in technological maturity (D4) reached values in a range of 510.4 mg/g for Crimson Seedless and 294.9 mg/g for Krissy™. These results agree with what was observed by Ortega-Regules et al. [42], who reported that different grape cultivars undergo different changes in their polysaccharide profiles during ripening and there is no change in arabinose concentration. Rhamnose, however, was more varied, either increasing, remaining constant, or decreasing slightly depending on the cultivar, which can explain the different evolution in certain fractions of polysaccharides.

At commercial harvest, the fraction of polysaccharides with the highest concentration corresponded to F2 (molecular mass between 500 and 35 KDa), followed by F3 (molecular mass from 35 to 5 KDa) and finally, F1 (molecular mass greater than > 500 KDa). This coincided with what was observed by Gil et al. [43], who reported that the total concentration of medium to low molecular weight polysaccharides in Cabernet Sauvignon cultivar grapes increased with greater grape maturity. Timco™ and Krissy™ presented a similar behavior (without statistical differences between D1 and D4). On the contrary, Crimson Seedless presented a decrease in this fraction.

### 2.4. Mechanical Behavior of Red Table Grape Varieties Crimson, Timco™, and Kryssy™ during Ripening and Extended Cold Storage Period

Texture is an important factor in determining the quality of table grapes for fresh consumption. Berry firmness is considered a measurement of its freshness [44].

Texture includes all physical properties perceived through contact that are related to deformation when a force is applied that can be objectively measured in terms of force, distance, and time [45]. These mechanical variables are correlated with several sensory attributes and thus indirectly with consumer acceptability of products [7,8].

Sensory characteristics such as skin thickness and friability and flesh firmness are suggested to differentiate commercial table grape cultivars [46]. Nevertheless, there are few descriptions of the physical–mechanical parameters of table grapes in the literature, and only a few reports are available on varietal differences in the mechanical properties of grape texture.

**Figure 1:**
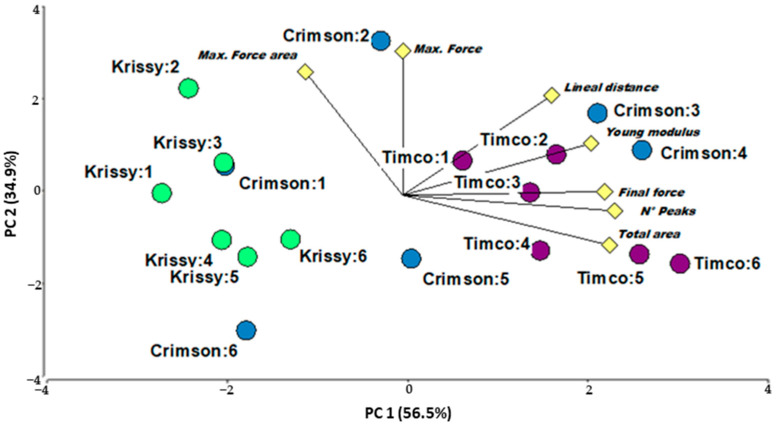
Two-dimensional plot of the two first principal components in PCA of the cultivars studied (Crimson Seedless, Timco™, Krissy™). Mechanical variables in yellow diamond (maximum force, maximum force area, lineal distance, Young’s modulus, final force, numbers of peaks, and total areas) and observation (variety:date) in dots.

**Figure 2:**
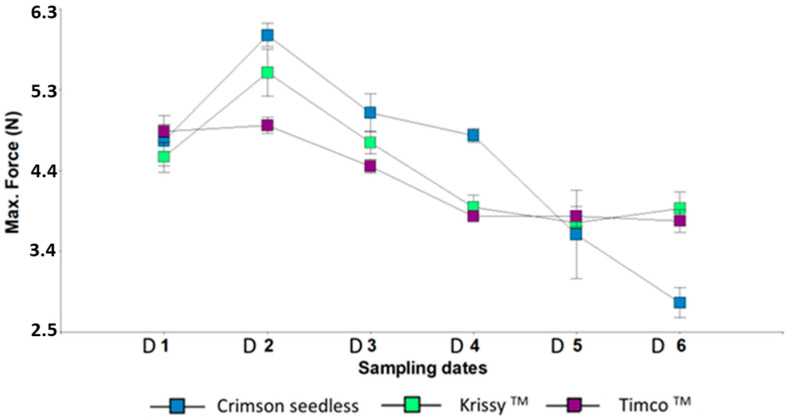
Maximum Force of three red table grapes at six different sampling dates. Standard error is shown for each parameter (*n* = 50). Sampling along harvest: D1, veraison; D2, 12 DAV (days after veraison); D3, 26 DAV; D4, 37 DAV. Sampling along storage: D5, 54 DOS (days of storage); D6, 108 DOS.

**Figure 3:**
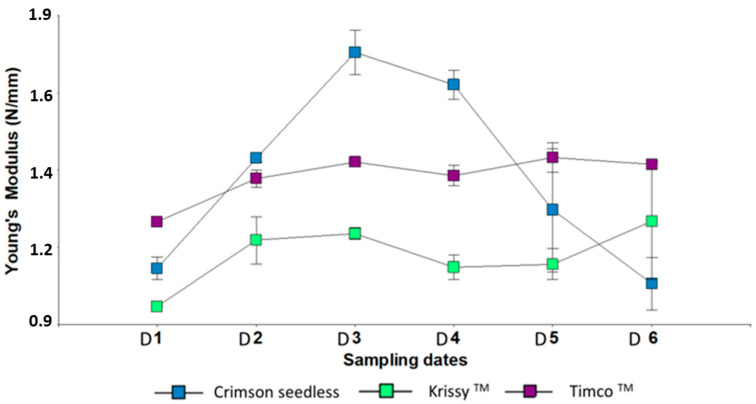
Young’s Modulus of three red table grapes in six different sampling dates. Standard error is shown for each parameter (*n* = 50). Sampling along harvest: D1, veraison; D2, 12 DAV (days after veraison); D3, 26 DAV; D4, 37 DAV. Sampling along storage: D5, 54 DOS (days of storage); D6, 108 DOS.

**Figure 4:**
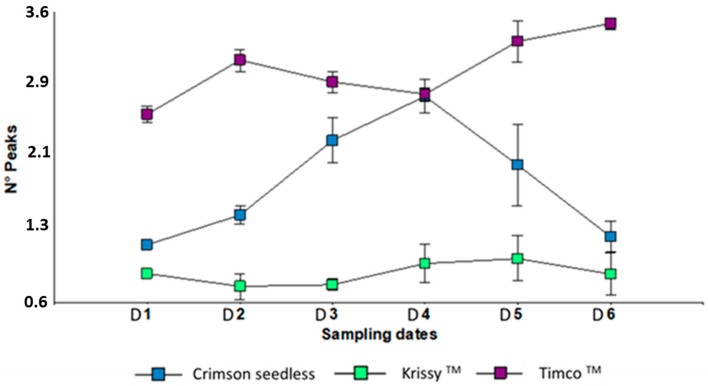
Peak numbers of three red table grapes in six different sampling dates. Standard error is shown for each parameter (*n* = 50). Sampling along harvest: D1, veraison; D2, 12 DAV (days after veraison); D3, 26 DAV; D4, 37 DAV. Sampling along storage: D5, 54 DOS (days of storage); D6, 108 DOS.

The results of the mechanical behavior of red table grape varieties Crimson, Timco™, and Kryssy™, during ripening and an extended cold storage period are presented below (Figure 1, Figure 2, Figure 3 and Figure 4).

Principal components analysis (PCA) was performed to better understand the differences among grapes according to the cultivar, physical, and mechanical parameters (Figure 1). From the loadings of selected variables (prerupture variable: Young’s modulus of elasticity of the skin; at rupture: maximum force, maximum force area; postrupture variables: number of peaks, final force, final force area, and lineal distance.). PC1 (56.50% total variance) was most highly correlated with skin mechanical properties such as lineal distance, Young’s modulus of the skin, final force, number of peaks, and total areas. PC2 (34.90% total variance) was most correlated with the maximum force and maximum force area.

Based on these results, the skin mechanical properties of table grapes were good parameters for differentiating varieties. Hence, they might be used to advantage as varietal markers, since these parameters are also little influenced by the ripening stage of the grape, as has been demonstrated for wine grapes [47].

The maximum force (firmness), began to decrease from the sampling date two for the three varieties. However, during the cold storage period after harvest, Timco™ and Kryssy™ maintained their firmness while it strongly decreased in Crimson Seedless (Figure 2). During the maturation period, these results coincide with those of Conner [48] for the germplasm of the Muscadine variety. According to the results of that author, the firmness was found to decline with increasing maturity and storage times, as was observed in the case of Crimson Seedless.

Young’s modulus is a measure of the elasticity (rigidity) of the skin. A low Young’s modulus represents a berry with low turgidity [48]. Timco™ and Kryssy™ tended to maintain their turgor, whereas Crimson Seedless was strongly affected by a long cold storage (Figure 3). Pericarp puncture parameters, particularly Young’s modulus and berry cohesiveness, were able to distinguish between Cabernet Franc grapes belonging to different vineyards in the Loire Valley, independently of the sugars accumulated in the berry pulp [49]. Therefore, the mechanical properties of Cabernet Franc grapes during the ripening process vary from vineyard to vineyard. From a physiological point of view, it is important to know that the texture of grapes, especially the skin texture, depends more on the region of origin than on the ripeness of the grapes at harvest [50]. In the present study, all the varieties were cultivated in the same locality, so the results correspond to the intrinsic characteristics of each variety in relation to the elasticity of the skin during maturation and in postharvest storage.

The puncture test is widely used to describe the texture (e.g., crunchiness and crispness features) in swollen foods [51,52]. This test measures the force required to push a punch or probe into a food. The features are (1) a force gauge; (2) penetration of the probe into the food causing irreversible crushing (that is, individual breakdown of the various cell walls that make up the product) or flow of the food; and (3) the depth of penetration is usually kept constant [53]. Therefore, the deformation behavior of crunchy/crispy foods is often studied as a function of time/displacement at lower deformation rates to study different possible fracture events separately [51,54]. This means that the force–deformation pattern for crispy products is characterized by a series of sharp force peaks corresponding to the rupture of individual cell walls [51,52]. The peaks represent microfractures associated with the microphenomena of sound nature [7,48]. The higher the number of peaks, the crispier the flesh is. For this work, Timco™ was the crispiest variety whereas Crimson Seedless’ peak counts decrease dramatically after cold storage (Figure 4).

### 2.5. Gene Expression

**Figure 5:**
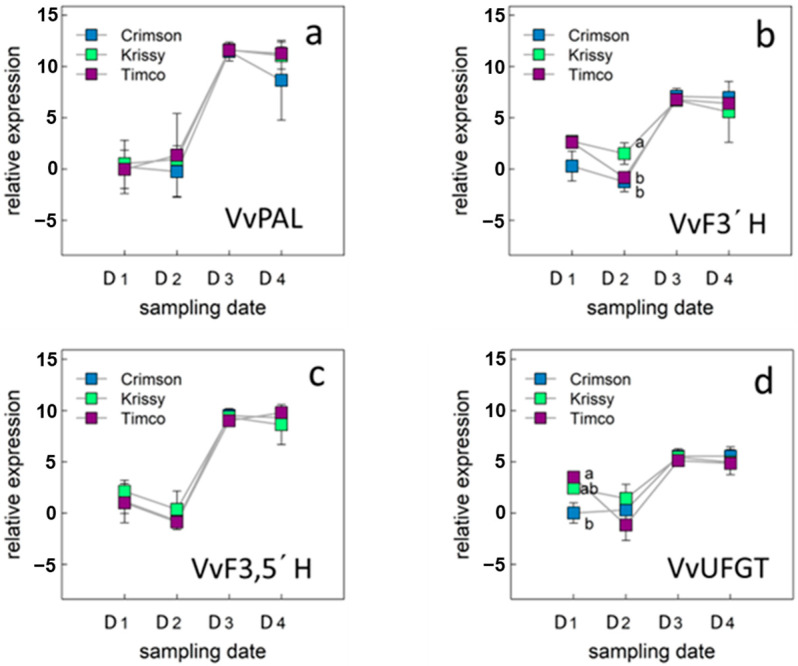
Expression level of genes involved in phenylpropanoid pathway: VvPAL (**a**), VvF3′H (**b**), VvF3,5′H (**c**), VvUFGT (**d**) and in table grape varieties Crimson Seedless, Krissy™, and Timco™. Vertical bars indicate the standard error of five biological replicates. Different letters indicate significant differences between treatments according to the Tukey test (*p* < 0.05). Sampling along harvest: D1, veraison; D2, 12 DAV (days after veraison); D3, 26 DAV; D4, 37 DAV.

**Figure 6:**
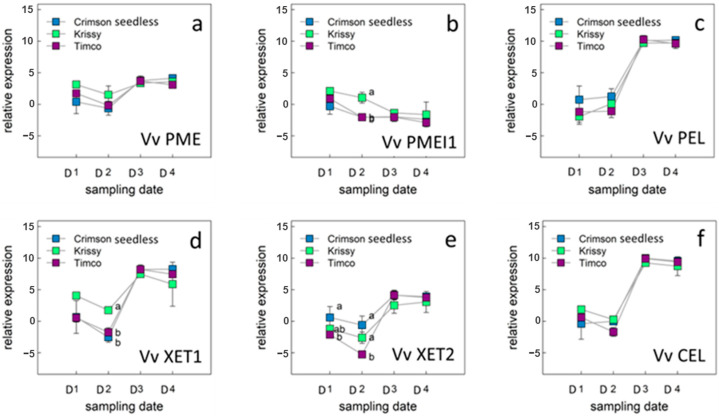
Expression level of genes related with the cell wall metabolism in berry skins: VvPME (**a**), VvPMEI1 (**b**), VvPEL (**c**), VvXET1 (**d**), VvXET2 I, and VvCEL (**f**) in table grape varieties Crimson Seedless, Krissy™, and Timco™. Vertical bars indicate the standard error of five biological replicates. Different letters indicate significant differences between treatments according to the Tukey test (*p* < 0.05). Sampling along harvest: D1, veraison; D2, 12 DAV (days after veraison); D3, 26 DAV; D4, 37 DAV.

To investigate the molecular nature of the metabolic shifts observed in grapes, we examined the expression of genes encoding key intermediates in central metabolic pathways only during the ripening period of table grapes (Figure 5 and Figure 6).

#### 2.5.1. Genes of the Phenylpropanoid Pathway of the Berry Skins

The expression profile of the phenylalanine ammonia lyase (VvPAL) gene that encodes the first enzyme of the phenylpropanoid pathway [55] was initially investigated (Figure 5a). The expression level of the VvPAL gene increases until sampling date 3 (D3) and then remains constant until commercial maturity (D4).

The behavior in the expression of the VvPAL gene, as well as that of the other three genes of the phenylpropanoid pathway studied during the ripening period of the berries, was similar for the three varieties studied (Figure 5a–d).

In table grapes, color is an important parameter from the consumer point of view and there are enzymes in the phenylpropanoid pathway directly related to the synthesis of anthocyanidins, precursors of anthocyanins, responsible for the color in red varieties. In the phenylpropanoid pathway, anthocyanidin synthase catalyzes the reaction of colorless leucoanthocyanidin to colored anthocyanidin, and hydroxylation at the 3′ and 5′ positions of the B ring is mediated by flavonoid 3′-hydroxylase (F3′H) and by flavonoid 3′,5′-hydroxylase (F3′,5′H), respectively. The hydroxyl groups on the B ring are known to affect the absorption spectra of anthocyanins, especially F3′,5′H is important for the formation of blue delphinidin, which affects the color of the fruit [56].

In this work, the relative expression of F3′H and F3′5′H increased until sampling date 3 and then kept constant, observing a slightly higher relative expression for VvF3,5′H with respect to VvF3′H (Figure 5b,c). Anthocyanin production in berries is primarily associated with two genes, VvUFGT and VvMYbA1, which encode the UDP-glucose: flavonoid-3-*O*-glucosyltransferase (UFGT) enzyme and an MYB-type transcription factor, respectively [55,57]. The UFGT enzyme catalyzes the glycosylation of anthocyanidins [58]. Since VvUFGT expression is closely associated with the activation of the anthocyanin signaling pathway [55,59], it can be used as a molecular marker to discriminate berry ripeness in terms of color development [59].

In this work, the expression pattern of VvUFGT was similar to that of the other genes studied for the three table grape varieties, but with a lower relative expression (Figure 5d). VvUFGT expression levels were related to tissue solute concentration (solute potential). In green skin tissues, low or undetectable levels of expression are associated with high tissue solute potentials, while increases in expression level correlate with decreases in solute potential [60]. The accumulation of sugars equates to a decrease in solute potential and is integral to regulating color development. The increase observed in the relative expression of the VvUFGT gene (Figure 5d) coincides with the increase in soluble solids in the berries (Table 1).

#### 2.5.2. Genes of the Cell Wall Metabolism of Berry Skins

Six genes were selected according to their biological role in the cell wall metabolism from the different profiles of expression (Figure 6a–f).

Grape harvest time is still more or less based on macroscopic parameters and is not always easy to determine. In addition to the concentration of sugars and phenols (such as anthocyanins responsible for skin color), it is important to consider berry softening. Table grapes must be soft enough to be accepted by consumers. Grape berry softening is a complex process involving subtle changes in the cell wall and requires only small amounts of enzymatic activity [40,61,62].

Analysis of cell wall polysaccharides during berry development and ripening revealed no significant changes in polysaccharide types [39]. However, marked changes in specific polysaccharides were observed, such as increased solubility of galacturonan and decreased in type I arabinogalactan (AGI) in the pectin polysaccharide fraction [3,61,63].

The primary reason that ripe fruits become softer is due to the degradation of the middle lamella of the cortical parenchyma cells. These cells are mostly comprised of pectins, which are composed of linear polygalacturonate chains that are intertwined with highly branched rhamnogalacturonan chains. Methyl and/or acetyl groups esterify some of the galacturonate residues. Depending on the source of the pectin, the degree of methylation and acetylation can vary greatly. On a dry weight basis, plant cell walls contain up to 7% of *O*-bound acetyl groups, which are largely linked to two polysaccharides, xylan and pectin. Esterases are required to remove this modification, which makes it easier for the polysaccharide chain to degrade even further [63,64].

Ripening of grape berries is associated with a decrease in the methylation levels of pectin. [65]. Pectins may be degraded by esterases such as pectin acetylesterase and pectin methylesterase (PME); glucosylhydrolases (polygalacturonases) and lyases (pectate lyse: PEL). PME activity increases during ripening in many species, but the activity is lower in grape than in other species [40,42]. PME is an enzyme of physiological relevance in plant metabolism, being involved in all processes requiring the remodeling of the plant cell wall, such as fruit ripening and cell extension. In this work, VvPME (Figure 5a) presented a similar behavior for the three varieties under study, with a slight increase in the relative expression during maturation (D1 to D4), but without significant differences between sampling dates. In general terms, the relative expression of VvPME is low, which would be related to what was observed by Nunan et al. [40], who, for berries of the Muscat Gordo blanco variety, point out that the activity of the PME enzyme was low and decreased after veraison.

Efficient regulation of PME (pectin methylesterase) activity is likely due to the presence of endogenous PMEIs (pectin methylesterase inhibitors), which belong to a large family of proteins encoded by multiple genes. Studies on VvP-MEI1 gene expression in various grape tissues suggest that this inhibitor plays an important role in regulating PME activity in the early stages of grape development and during flowering [65,66].

In this work, (Figure 5b) the relative expression of the VvPME1 decreased from the early stages of the maturation process until commercial harvest, especially in the Timco™ and Krissy™ varieties, with a significant difference in Crimson Seedless in D2, which presented the highest values of VvPEMI1 relative expression in that sampling date. From D3 to the date of technological maturity (D4), the three varieties did not present differences in the relative expression of VvPMEI1. This agrees with Lioneti et al. [65], who observed that VvPMEI1 mainly expressed in the early phase of berry development, possibly participating in the modulation of pectin methylesterification at this specific stage.

PEL activity is difficult to detect in fruits. Previous studies in grape berries have reported a relation between this enzyme and fruit softening during ripening [40,67]. Pectin degradation by pectate lyase occurs by a β-elimination reaction, in contrast to the hydrolytic mechanism of polygalacturonases. Pectate lyase catalyzed the cleavage of de-esterified pectin. Thus, pectin methylesterase and acetylesterase activity must either precede the activity of pectate lyase or occur at the same time. In this work, the VvPEL gene’s relative expression was higher compared to the VvPEL during the four sampling dates during the ripening of the berries. For all the varieties under study, an increase in the relative expression of both genes was observed and was later stabilized in the advanced stages of maturity, between sampling dates D3 and D4. According to other authors [40,68], PEL transcripts are mostly abundant at veraison and after veraison, in agreement with the results presented here. On the contrary, Glissant et al. [61] point out that PL transcripts are less expressed after veraison and it expresses in berries from pea size until veraison, showing the highest level of expression at veraison and the lowest just before. Vargas et al. [69] observed several polymorphisms in the VvPel gene that may explain the different results obtained by different authors in relation with the VvPel expression during grape berry ripening.

Cellulose content is stable or slightly increased [66,70], but the xyloglucan portion of hemicellulose is partially depolymerized in some species during fruit ripening [71]. Xyloglucan endotransglycosylases (XET) belongs to a family of enzymes that mediate hemicellulose reassembly. According to Schlosser et al. [72], xyloglucan endotransglycosylase transferase (XET) expression in exocarp and mesocarp was severely downregulated during phase I of grape berry growth, but upregulated again during phase II and into phase III for Cabernet Sauvignon berries. In this work, VvXET1 presented a higher relative expression than VvXET2, but both presented a similar behavior during ripening. The relative expression of VvXET1 and VvXET2 initially decreased from veraison (D1) until the second sampling date, and then increased until D3, remaining stable until technological maturity (D4). These results are consistent with those of Glisant et al., who described four XET isoforms in grapes, three of which were expressed mainly at the beginning of the ripening process, semi-green, or before veraison [61]. A fourth isoform peaked after veraison.

Plants encode cellulases [EC] that catalyze the cleavage of internal 1,4-α-glucosidic bonds in cellulose, which makes up their tissues. Plant cellulases have been suggested to be essential for many aspects of plant development, including abscission and fruit softening [73]. According to Schlosser et al. [72], endo-(1→4)-glucanase (CEL) expression was more pronounced in the exocarp and mesocarp tissues during stage I and III of berry development than in stage II and later stage III parts. This agrees with the results obtained in this work in which a similar behavior of VvCEL was observed for the three varieties studied. In the middle of the ripening process (D2 to D3), an important increase in the relative expression of this gene was observed, with low and stable values of relative expression in the early stages of maturation between D1 and D2 and high and stable values between D3 and D4.

## 3. Materials and Methods

### 3.1. Plant Material

Information on the variety origins, table grapes’ vineyard cultivation, and trials is described in detail in a previous work [74].

Grapes from Crimson Seedless, Krissy™, and Timco™ cultivars (*Vitis vinifera* L.) were collected at four moments: veraison (D1); 12 days after veraison (D2); 26 days after veraison (D3); 37 days after veraison (D4; corresponding to technological maturity). At each sampling date, 90 berries with pedicel per replicate were harvested: 50 berries were selected and kept at −20 °C to measure pH, total acidity, soluble solids, phenolic composition, polysaccharidic composition, and weight. On the other hand, 30 berries were used for texture analyses, equatorial diameter, and length. The 10 remaining berries were stored immediately after harvest at −80 °C until gene expression analysis.

After harvesting at technology maturity, the grape bunches from the commercial vineyard were packaged conventionally according to commercial practices and were stored. The clusters were packaged in individual perforated polyethylene bags (6.5% ventilation area); a total of 8 clusters per 8.2 kg wooden box were packed in a second polyethylene bag with a ventilation area of 0.3% or 2.0%, including SO_2_ generating mat. The boxes were stored for 75 days at 1–0 °C and >85% relative humidity. During storage, samples of Crimson Seedless, Krissy™, and Timco™ varieties were collected on two dates: 54 days in storage (D5); 75 days in storage (D6) [74]. For each cultivar, three replicates (boxes) were considered at each sampling date. A total of 50 berries from different bunches in each case were analyzed for general chemical analyses (pH, titratable acidity, and soluble solids), and 30 stemmed berries were stored at −20 °C for chromatographic analysis.

### 3.2. Chemicals

Standards of (−)-epicatechin and (+)-catechin (purity > 98%), gallic acid (purity > 97%), and malvidin-3-glucoside (purity > 90%); 0.45 µm pore size membranes and methylcellulose (1500 cP viscosity at 20 g/L) were purchased from Sigma Chemical Co. (St. Louis, MO, USA). Polyethylene membranes with a pore size of 0.22 μm were purchased from EMD Millipore (Burlington, MA, USA). HPLC grade ethyl acetate, diethyl ether, hydrochloric acid, sulfuric acid, acetonitrile, acetic acid, formic acid, methanol, potassium metabisulfite, sodium hydroxide, and vanillin (990 g/L) were acquired from Merck (Darmstadt, Germany). All reagents were of analytical grade or higher. Phosphate buffer (pH 7) was purchased from Mallinckrodt Baker (Philipsburg, PA, USA). For column calibration in gel permeation chromatography (GPC) analysis, *Leuconostoc* dextran analytical standards were used (dextran 5000, 12000, 25000, 50000, 80000, 150000, 270000, 410000, and dextran 670000) (Sigma-Aldrich Co., St. Louis, MO, USA). Nitrogen was provided by INDURA S.A. (Santiago, Chile).

### 3.3. General Chemical Composition

The soluble solid concentration, pH, and titratable acidity were measured by employing the analytical methods recommended by the International Organization of Vine and Wine [75]. The pH was measured as a function of hydrogen ion concentration by potentiometry (Model Seven Compact S220, Mettler-Toledo Intl. Inc., Columbus, OH, USA), and titratable acidity was measured with 0.1 N NaOH. Lastly, the weight of 50 berries was registered in grams and length and equatorial diameter in millimeters.

### 3.4. Phenolic Composition and Individual Anthocyanins Analyses

Sample preparation: The skins were removed from the grapes, washed with distilled water, and dried on absorbent paper. An amount of 1 g of fresh skin was minced and extracted with 10 mL of acidified methanol (MeOH:HCl 99:1) by shaking on a mechanical shaker at 4 °C for 24 h in the dark. After extraction, the extract was filtered through a 0.45 µm syringe filter and stored at −18 °C until analysis.

Total phenolic concentration was determined by UV absorption spectrophotometry at 280 nm using gallic acid as standard [76]. Total tannin content was determined by the methylcellulose method [77] using (−)-epicatechin as standard. Anthocyanin content (expressed as mg/L malvidin-3-glucoside) was measured by SO_2_ bleaching [76]. Absorption measurements were performed using a Hewlett-Packard UV-Vis 1700 Pharmaspec spectrophotometer (Shimadzu, Kyoto, Japan). Antioxidant capacity was determined by using a Perkin Elmer 2030 VICTOR X2 spectrofluorometer according to Nicolosi et al. [33]. Results were expressed as Trolox equivalent antioxidant capacity (TEAC).

The content of anthocyanins in extracts was evaluated according to the methodology described by Peña-Neira et al. [78] and Cejudo-Bastante et al. [79]. The chromatographic analysis was performed by using an 1100 Series HPLC system (Agilent Technologies, Santa Clara, CA, USA) consisting of a G1329A autosampler, a G1311A a quaternary pump, a G1315B photodiode array detector (DAD), and G1379A degasser. The chromatographic conditions were detailed by Peña-Neira et al. [78]. Prior to direct injection of the methanol extract from the grape skins, the samples were filtered through a membrane with a pore size of 0.22 μm. All analyses were performed in triplicate. A wavelength of 520 nm was used for quantification by comparing the area and retention time with the malvidin-3-glucoside standard.

### 3.5. Polysaccharide Analysis

The procedure for the extraction of soluble polysaccharides from grape skins was carried out as described by Gil-Cortiella and Peña-Neira [80]. The quantification of the polysaccharides fractions was carried out using dextrans to assess the molecular weight of polymeric fractions and pectins as the external standard to quantify the polysaccharides using previously described developed and validated methodologies [79,80].

The analysis of soluble polysaccharides was carried out by using a high-performance size exclusion chromatography–refractive index detection (HPSEC-RID) system (Agilent 1260 Infinity Series liquid chromatograph; Santa Clara, CA, USA) consisting of a G1329A autosampler, a G1362A refractive index detector, a G1315D diode array detector, a G1311B quaternary pump, a G1316A column oven. The chromatograph was connected to an Agilent Chem Station data processing station (version B.04.03).

### 3.6. Texture Analysis

Puncture test was performed with 2.0 (P2) mm diameter probe using a TA-XT Plus (Stable Micro Systems, Surrey, UK) texture analyzer. For texture analysis, a set of 30 berries were randomly sampled. Each one of the intact grape berries was individually compressed, and the instrumental mechanical parameters were measured or calculated. Curve force versus distance was calculated by using mechanical variables of pre- and postrupture (i) prerupture variable: Young’s modulus; (ii) at rupture: maximum force, maximum force area; (iii) postrupture variables: number of peaks, final force, final force area, and lineal distance). Before each test session, the instrument was calibrated for force and distance [7,45].

### 3.7. Analysis of Gene Expression

#### 3.7.1. Primer Design

Primers related to flavonoid biosynthesis and cell wall metabolism were designed with Primer Premier 6 (Premier Biosoft, Palo Alto, CA, USA) and their specificity was assessed with the software Primer Blast from the NCBI website. The primers summarized in Table 4 related to cell wall metabolism correspond to cellulase (CEL), pectate lyase (PEL), xyloglucan endotransglycosilase 1 (XET1), xyloglucan endotransglycosilase 2 (XET2), pectin methyl esterase (PME), PME inhibitor (GRIP28); for flavonoid biosynthesis, they are flavonoid 3′ hydroxylase (F3′H), flavonoid 3′ 5′ hydroxylase (F3′5′H), and UDP glucose:flavonoid 3-*O*-glucosyltransferase (UFGT) and the reference genes Actin, GADPH, and AIG1, the latter suggested as a putative reference gene for table grapes [81].

#### 3.7.2. RNA Extraction from Grape Skin and cDNA Synthesis

RNA extraction was performed from grapes previously stored at −80 °C. The skins were peeled with fresh, sterile razor blades, immersed in liquid nitrogen in a mortar, and immediately pulverized with a pistil until a fine powder was achieved. RNA was extracted following the sodium perchlorate method [55], with some modifications. The still cold fine powder was transferred to a 50 mL falcon tube and slowly added to 15 mL of the extraction buffer which contained sodium perchlorate 5 M, Tris 0.3 M pH 8.3, SDS 1%, PEG 20000 2%, PVPP 8.5%, and 2-mercaptoethanol 2%. The extract was vortexed for 45 s and stored at −20 °C for 45 min. The extract was then passed through a syringe containing glass wool and a 0.45 um PVDF filter at the base by centrifuging at 1500× *g* for 5 min at 4 °C. The eluate was then vortexed with 1.5 vol. of 100% cold ethanol, allowed to precipitate for 30 min at −20 °C, and then centrifuged at 1500× *g* for 45 min at 4 °C. Then, the supernatant was discarded and 2 mL of Tris 10 mM with EDTA 1 mM pH 7.5 was added to the precipitate. The aqueous phase was recovered after each addition of 1 vol. of phenol, chloroform, and isoamyl alcohol (25:24:21) and 1 vol. of chloroform: isoamyl alcohol (24:1) to the above solution. To the aqueous solution was added 0.1 vol of sodium acetate 3 M pH 5.2 and 2 vol. of 80% cold ethanol and the genetic material was allowed to precipitate for 2 h at −20 °C and then centrifuged at 12,500× *g* for 30 min at 4 °C. Subsequently, the supernatant was discarded, then 1 mL of 80% cold ethanol was added to the precipitate which was then sedimented, rinsed, and dried. Subsequently, the supernatant was discarded, 1 mL of cold 80% ethanol was added to the resulting precipitate, and then centrifuged at 10,000× *g* for 10 min at 4 °C, and then the supernatant was discarded again and allowed to dry. The pellet was resuspended in nuclease-free water, 0.25 vol of LiCl_2_ 10 M was added, and the RNA precipitate was kept overnight at 4 °C. The pellet was cleaned twice with 80% cold ethanol, resuspended with 40 μL nuclease-free water, and treated with recombinant DNase I (Roche, Mannheim, Germany), following the manufacturer’s instructions. Quality and quantity of resultant RNA was assessed through electrophoretic bleach-gel technique [82] and spectrophotometry (OD 260/280). The cDNA synthesis was performed with 1 ug of RNA, which was then reverse transcribed using the SuperScript™ IV First-Strand cDNA Synthesis Reaction kit (Thermo Fisher Scientific, Carlsbad, California, United States), following the manufacturer’s protocol.

#### 3.7.3. Gene Expression Analysis by qPCR

Gene expression analysis was performed by the Lightcycler™ 96 system (Roche Diagnostics, Mannheim, Germany) and using FastStart Essential DNA Green Master kit (Roche Diagnostics, Mannheim, Germany), following the manufacturer’s protocols. The mixture for the qPCR reaction was 0.5 µM of each primer, a 1:50 dilution of the cDNA, and the master mix in a final reaction volume of 20 µL. Amplification curves analysis was performed by the LightCycler^®^ 96 software (Roche Diagnostics, Mannheim, Germany) and the purity of the amplified products was confirmed by melting curve analysis. Each sampling date had 3 biological replicates and 3 technical replicates for each grape variety. To obtain the relative expression of each gene, five 10-fold serial dilutions were assessed to calculate the amplification efficiency according to (1), where E is the efficiency for each primer and m is the slope between serial dilutions and the threshold cycle (Cq).

(1) $${E=10}^{\left(-\frac{1}{m}\right)}-\;1$$

The efficiencies for each primer were summarized in Table 4, and these values were used to obtain the relative expression of each gene of interest (GOI) with respect to their reference genes (R) following the expression (2). To obtain the relative expression and follow their evolution through time, the Cq of GOI and reference genes were referenced to the Cq mean value of each gene from the Crimson variety at the first sampling date.

(2) $$\mathrm{relative}\;\mathrm{expression}=\frac{E_{\mathrm{GOI}}^{-{(\mathrm{Cq}}_{\mathrm{GOI}}-{\mathrm{Cq}}_{\mathrm{GOI}\mathrm{Cal}})}}{\sqrt[{n}]{\prod_{i=1}^{n}E_{\mathrm{Ri}}^{-{(\mathrm{Cq}}_{\mathrm{Ri}}-{\mathrm{Cq}}_{\mathrm{Ri}\mathrm{Cal}})}}}$$

### 3.8. Statistical Analyses

Data analysis was performed using InfoStat (version 2017p, FCA-Universidad Nacional de Córdaba, Argentina). Means were compared using ANOVA and post hoc test (Tukey) (α = 0.05). All tests met the assumption of residual normality. Gene expression analyses for each sampling date were evaluated by ANOVA analysis, testing the assumptions of normality and homogeneity of variance. Means were subjected to post hoc Tukey analysis when *p*-values were less than 0.05.

## 4. Conclusions

Determining the optimum harvest time is a significant factor affecting the quality of table grapes. Monitoring the evolution of grapes’ physicochemical properties, phenolic, and polysaccharidic composition and texture properties during ripening could be a valuable tool for determining the optimum harvest time to ensure optimal postharvest conditions and market acceptability. The market is looking for grapes with a superior size, condition, and taste and has been introducing new varieties from several national and international breeding programs, such as Timco™ and Krissy™, that are replacing popular grape varieties, such as Crimson Seedless. According to the results, phenolic compounds from skins were present in very different proportions among the varieties studied. The total anthocyanins responsible for the color of the skins increased during ripening and the majority individual anthocyanins in the three varieties was peonidin-3-glucoside, followed by malvidin-3-glucoside. The phenolic compounds presented a different behavior (decreasing or increasing) during postharvest. The total skin soluble polysaccharides decreased during ripening and postharvest in Crimson Seedless and Kryssy™ and remained constant from technological maturity to postharvest storage in Timco™. In all varieties, the majority soluble polysaccharide fraction was that with a molecular mass between 500 and 35 KDa (F2). The skin mechanical properties of table grapes were good parameters for differentiating varieties, with better results for the new varieties Timco™ and Kryssy™, compared to the traditional variety Crimson Seedless, especially postharvest. For this work, Timco™ was the crispiest variety whereas Crimson Seedless’ peak counts decreased dramatically after cold storage. Timco™ and Kryssy™ tended to maintain their turgor, whereas Crimson was strongly affected by a long cold storage. The firmness began to decrease early during ripening for the three varieties; however, at the cold storage period, Timco™ and Kryssy™ maintained their firmness while it strongly decreased in Crimson. The cell wall metabolism gene expression profiles followed similar trends in exocarp tissues throughout berry development in all the varieties studied. Similar results were observed for genes involved in the phenylpropanoid pathway. These results may position the Timco™ and Kryssy™ varieties as a very good alternative and competitor to Crimson Seedless, which is currently one of the table grape cultivars in Chile with a high commercial demand.

## Figures and Tables

**Table 1:** Data of sampling, the berry weight, equatorial diameter, length, and technological maturity parameters of grapes from cultivars Crimson Seedless, Timco™, and Krissy™ during ripening and postharvest storage.

Variable	Cultivar	Period before Harvest				Period Postharvest	
Sampling Dates:		30 January 2018 (D1)	12 February 2018 (D2)	26 February 2018 (D3)	9 March 2018 (D4)	2 May 2018 (D5)	25 June 2018 (D6)
Weight of 50 berries (g)	Crimson	244.8 ± 18.5 a	264.6 ± 22.4 ab	296.4 ± 25.3 b	294.0 ± 37.9 b	262.5 ± 22.4 ab	262.7 ± 27.8 ab
	Timco™	420.8 ± 21.2 a	471.3 ± 24.6 ab	493.2 ± 13 ab	522.0 ± 60.4 b	527.8 ± 57.5 b	526.2 ± 29.7 ab
	Krissy™	331.7 ± 14.7 a	426.7 ± 39.4 ab	485.1 ± 28.3 b	432.8 ± 27.2 ab	420.1 ± 78.5 ab	457.6 ± 90.5 b
Length (mm)	Crimson	17.58 ± 0.55 a	18.25 ± 0.25 a	18.70 ± 0.81 a	18.27 ± 0.84 a	17.04 ± 0.34 a	16.64 ± 0.56 a
	Timco™	21.91 ± 0.36 a	22.71 ± 0.56 a	22.82 ± 0.33 a	22.68 ± 0.93 a	22.19 ± 0.67 a	21.96 ± 1.1 ab
	Krissy™	20.87 ± 0.36 a	22.90 ± 0.90 b	23.83 ± 0.33 b	22.42 ± 0.39 b	21.75 ± 1.47 b	21.50 ± 2.11 b
Equatorial diameter (mm)	Crimson	22.52 ± 0.53 a	24.11 ± 1.16 b	25.20 ± 0.49 b	24.72 ± 0.50 b	24.06 ± 1.02 b	23.37 ± 1.19 ab
	Timco™	26.73 ± 0.67 a	28.25 ± 0.55 b	28.77 ± 0.47 b	30.53 ± 1.17 b	30.14 ± 1.73 b	29.67 ± 0.58 b
	Krissy™	20.85 ± 1.06 a	25.66 ± 0.84 b	25.58 ± 0.67 b	24.91 ± 0.84 b	24.72 ± 1.25 b	23.07 ± 2.62 ab
Total soluble solids (°Brix)	Crimson	13.47 ± 0.31 c	15.13 ± 0.12 cb	16.87 ± 0.12 b	18.73 ± 0.31 ab	20.20 ± 0.35 ab	21.73 ± 0.64 a
	Timco™	13.47 ± 0.23 c	16.20 ± 0.20 bc	17.27 ± 0.31 b	18.73 ± 0.23 ab	19.40 ± 0.20 a	21.40 ± 0.35 a
	Krissy™	13.60 ± 0.20 c	16.07 ± 0.31 bc	18.33 ± 0.12 bc	19.40 ± 0.53 b	20.87 ± 1.01 ab	22.07 ± 0.31 a
pH	Crimson	2.90 ± 0.01 a	3.18 ± 0.04 a	3.16 ± 0.02 a	3.35 ± 0.52 a	3.50 ± 0.02 a	3.54 ± 0.04 a
	Timco™	2.92 ± 0.03 a	3.21 ± 0.06 a	3.26 ± 0.06 b	3.36 ± 0.02 a	3.50 ± 0.02 a	3.56 ± 0.05 a
	Krissy™	2.90 ± 0.02 a	3.17 ± 0.07 a	3.30 ± 0.03 b	3.40 ± 0.04 a	3.50 ± 0.06 a	3.54 ± 0.01 a
Titratable acidity (g H_2_SO_4_/L)	Crimson	6.35 ± 0.10 ab	5.97 ± 0.26 b	3.68 ± 0.21 b	3.22 ± 0.14 c	3.95 ± 0.24 b	3.98 ± 0.11 b
	Timco™	6.62 ± 0.60 a	5.36 ± 0.34 a	3.54 ± 0.10 a	3.74 ± 0.07 a	4.32 ± 0.29 a	4.03 ± 0.10 a
	Krissy™	8.20 ± 0.02 b	7.15 ± 0.25 a	4.32 ± 0.07 ab	4.27 ± 0.22 bc	5.04 ± 0.21 b	4.8 ± 0.04 a

Different letters within a row indicate statistical differences among sampling points (*p* < 0.05, Tukey post hoc test) for the same variable and cultivar. Sampling along harvest: D1, veraison; D2, 12 DAV (days after veraison); D3, 26 DAV; D4, 37 DAV. Sampling along storage: D5, 54 DOS (days of storage); D6, 108 DOS.

**Table 2:** Total phenols, anthocyanins, tannins, antioxidant capacity, and main individual anthocyanins in skins in Crimson Seedless, Timco™, and Krissy™ cultivars during ripening and postharvest storage.

Variable ^1^	Cultivar	Period before Harvest				Period Postharvest	
Sampling Dates:		30 January 2018 (D1)	12 February 2018 (D2)	26 February 2018 (D3)	9 March 2018 (D4)	2 May 2018 (D5)	25 June 2018 (D6)
Total phenols (mg/g FW)	Crimson	1.32 ± 0.09 b	1.57 ± 0.09 c	1.52 ± 0.18 c	1.51 ± 0.23 c	1.23 ± 0.03 a	1.29 ± 0.28 ab
	Timco™	0.75 ± 0.10 a	0.89 ± 0.07 b	0.78 ± 0.41 ab	0.75 ± 0.06 ab	0.86 ± 0.16 b	0.89 ± 0.15 b
	Krissy™	1.45 ± 0.12 b	1.46 ± 0.12 b	1.33 ± 0.11 a	1.44 ± 0.13 b	1.87 ± 0.54 bc	1.90 ± 0.17 c
Total anthocyanins (mg/g FW)	Crimson	0.40 ± 0.03 a	0.81 ± 0.07 c	0.78 ± 0.09 c	0.75 ± 0.15 c	0.81 ± 0.04 c	0.65 ± 0.12 b
	Timco™	0.33 ± 0.09 b	0.34 ± 0.09 b	0.33 ± 0.16 b	0.38 ± 0.05 c	0.29 ± 0.13 a	0.29 ± 0.09 a
	Krissy™	0.34 ± 0.03 a	0.55 ± 0.10 b	0.63 ± 0.06 c	0.65 ± 0.07 c	0.74 ± 0.27 c	0.57 ± 0.03 b
Total tannins (mg/g FW)	Crimson	6.67 ± 0.25 b	5.77 ± 0.44 b	5.36 ± 0.98 ab	5.47 ± 1.33 ab	4.75 ± 0.66 a	4.81 ± 1.11 ab
	Timco™	5.61 ± 3.83 b	3.24 ± 0.69 a	5.87 ± 0.62 b	3.48 ± 0.63 a	4.38 ± 0.85 a	5.10 ± 0.63 a
	Krissy™	8.96 ± 2.94 ab	6.57 ± 0.95 b	6.22 ± 0.55 b	6.57 ± 0.67 b	9.17 ± 2.96 ab	9.53 ± 1.63 a
Antioxidant activity (ORAC) (mmol/g TE FW)	Crimson	4188 ± 195 a	5569 ± 366 b	5396 ± 670 b	4627 ± 169 ab	5477 ± 195 b	4450 ± 212 ab
	Timco™	1180 ± 161 a	2368.33 ± 484 b	2463 ± 190 b	2453 ± 289 b	3105 ± 181 c	2894 ± 286 bc
	Krissy™	3428 ± 262 a	4710 ± 327 b	4713 ± 146 b	3821 ± 145 ab	3105 ± 181 a	3088 ± 223 a
Delphinidin-3-glucoside (μg/g FW)	Crimson	ND	11.1 ± 0.10 a	12.2 ± 0.30 a	13,4 ± 0.50 a	13.1 ± 0.20 a	12.6 ± 0.30 a
	Timco™	ND	ND	ND	ND	ND	ND
	Krissy™	10.10 ± 0.20 b	7.20 ± 0.30 a	12.01 ± 0.10 c	10.41 ± 0.10 b	10.31 ± 0.20 b	9.21 ± 0.12 b
Cyanidin-3-glucoside (μg/g FW)	Crimson	10.01 ± 0.31 a	18.20 ± 0.31 b	20.12 ± 0.27 b	21.20 ± 0.17 b	19.35 ± 0.21 b	19.21 ± 1.02 b
	Timco™	20.12 ± 1.01 a	19.17 ± 1.01 a	20.67 ± 0.61 a	19.58 ± 1.17 a	20.02 ± 1.13 a	19.00 ± 1.01 a
	Krissy™	20.02 ± 1.00 a	19.41 ± 1.63 a	30.03 ± 3.44 b	51.61 ± 4.83 bc	50.37 ± 5.16 bc	56.10 ± 7.12 c
Petunidin-3-glucoside (μg/g FW)	Crimson	ND	10.01 ± 0.90 a	10.71 ± 1.21 a	11.09 ± 1.36 a	10.21 ± 1.98 a	10.11 ± 0.96 a
	Timco™	ND	ND	ND	ND	ND	ND
	Krissy™	11.07 ± 0.71 a	20.12 ± 3.01 b	20.42 ± 1.97 b	40.25 ± 2.58 c	17.35 ± 3.13 b	15.01 ± 2.08 b
Peonidin-3-glucoside (μg/g FW)	Crimson	70.16 ± 4.14 a	110.41 ± 5.29 ab	113.71 ± 4.86 b	249.97 ± 6.19 d	222.39 ± 4.02 c	222.95 ± 5.06 c
	Timco™	40.27 ± 4.75 a	80.62 ± 4.99 b	100.96 ± 6.07 c	120.82 ± 5.54 cd	140.76 ± 7.21 d	110.91 ± 6.95 cd
	Krissy™	70.17 ± 4,29 a	80.23 ± 7.03 a	140.86 ± 8.32 b	210.88 ± 6.97 c	223.83 ± 8.23 c	212.20 ± 6.01 cd
Malvidin-3-glucoside (μg/g FW)	Crimson	113.19 ± 7.11 a	119.41 ± 4.28 a	127.12 ± 6.02 a	147.44 ± 6.92 b	172.17 ± 6.81 c	116.19 ± 6.54 a
	Timco™	20.92 ± 3.81 a	30.86 ± 5.51 b	38.03 ± 5.86 b	39.03 ± 2.42 b	27.32 ± 3.81 b	31.43 ± 4.42 b
	Krissy™	31.43 ± 4.31 a	42.74 ± 4.62 a	91.29 ± 5.71 b	88.28 ± 6.53 b	44.24 ± 5.71 a	51.45 ± 6.31 ab

^1^ Different letters within a row indicate statistical differences among sampling points (*p* < 0.05, Tukey post hoc test) for the same variable and cultivar. Total phenols are expressed as Gallic acid equivalents. Total tannins are expressed as Catechin equivalents. Anthocyanins are expressed as malvidina-3-glucoside equivalents. FW means fresh weight. Sampling along harvest: D1, veraison; D2, 12 DAV (days after veraison); D3, 26 DAV; D4, 37 DAV. Sampling along storage: D5, 54 DOS (days of storage); D6, 108 DOS.

**Table 3:** Skin soluble polysaccharides (mg of pectin/g of skins) according to molecular mass distribution: F1 > 500 KDa, F2: 500–35 KDa, F3: 35–5 KDa.

Variable ^1^	Cultivar	Period before Harvest				Period Postharvest	
Sampling Dates:		30 January 2018 (D1)	12 February 2018 (D2)	26 February 2018 (D3)	9 March 2018 (D4)	2 May 2018 (D5)	25 June 2018 (D6)
F1 (mg/g of skins)	Crimson	134.1 ± 12.4 bc	161.1 ± 12.2 bc	164.4 ± 109.5 c	81.3 ± 4.8 ab	79 ± 52.8 ab	25.1 ± 9.8 a
	Timco™	ND	ND	ND	13 ± 1.4 a	20.5 ± 4.2 ab	22.3 ± 5.4 b
	Krissy™	23.2 ± 4.9 ab	24.7 ± 16.1 b	16.6 ± 3.8 ab	15.7 ± 1.8 ab	9.4 ± 1.2 a	12.9 ± 4 ab
F2 (mg/g of skins)	Crimson	354.5 ± 35.9 c	374 ± 17.1 c	308.3 ± 21.6 c	281.7 ± 16.7 bc	127.3 ± 44.6 a	163.1 ± 4 ab
	Timco™	179.7 ± 38.4 b	118.8 ± 14.7 a	113.5 ± 43.9 a	209.5 ± 8.2 b	209.2 ± 36.9 b	206.1 ± 33.9 b
	Krissy™	153.6 ± 26.9 ab	123.7 ± 26.6 ab	129.4 ± 11.0 ab	159.8 ± 24.7 b	113.7 ± 14.6 a	147.1 ± 24.5 ab
F3 (mg/g of skins)	Crimson	266.7 ± 3.1 c	297.8 ± 29.9 cd	320.4 ± 10 d	147.4 ± 15.7 b	71.8 ± 28 a	101.6 ± 10.7 a
	Timco™	173.9 ± 82.4 b	89.1 ± 6.7 a	59.7 ± 29.6 d	98.7 ± 15.5 a	97.4 ± 7.7 a	109.9 ± 14.7 ab
	Krissy™	227.9 ± 40.4 c	185.4 ± 79.3 bc	116.9 ± 25.0 ab	119.4 ± 16.3 ab	84.7 ± 11.2 a	116.9 ± 16.4 ab
Total polysaccharides (mg/g of skins)	Crimson	755.3 ± 30.3 c	832.8 ± 50.1 c	793.1 ± 335.4 c	510.4 ± 14.4 b	278.1 ± 119.1 a	289.8 ± 23.1 a
	Timco™	353.6 ± 118.9 c	207.9 ± 12.2 ab	173.2 ± 73.5 a	321.2 ± 11.9 bc	327.1 ± 40.8 bc	338.3 ± 53.8 c
	Krissy™	404.7 ± 59.3 c	333.8 ± 83.6 bc	262.8 ± 32 ab	294.9 ± 38.7 ab	207.8 ± 24.7 a	276.9 ± 42.7 ab

^1^ Different letters within a row indicate statistical differences among sampling points (*p* < 0.05, Tukey post hoc test) for the same variable and cultivar. Results are expressed as pectin equivalents. Sampling along harvest: D1, veraison; D2, 12 DAV (days after veraison); D3, 26 DAV; D4, 37 DAV. Sampling along storage: D5, 54 DOS (days of storage); D6, 108 DOS.

**Table 4:** Summary of primers used for real-time PCR analysis in grape skins.

Gene Abv	Accession	Sense	Sequence (5′→3′)	Amplicon (bp)	Tm (°C)	Efficiency
CEL	AY043236.1	Forward	TTCTCCCAAGCCCAGTACACCC	147	61.0	1.81
		Reverse	AGTTCCACCGCAGTTCACAACT			
PEL	NM_001281122.1	Forward	GTGGAGGCATTGGAACTGGAGA	121	60.9	1.78
		Reverse	TGGTCTGGCACTCAAGCTGGAA			
XET1	AY043237.1	Forward	AGCCTCTGGAATGCGGATGACT	123	61.1	1.96
		Reverse	TGTGCTTGTGGAGGTGGAAGTG			
XET2	AY043238.1	Forward	AGCCTGTGGAATGCGGATGACT	121	61.45	1.98
		Reverse	CCACTGAAGCCTCACACCCATC			
PME	NM_001281162.1	Forward	GTGATGCCACGGTGGTCTTCCA	85	62.8	1.96
		Reverse	CCTTGGGCGGTGATGGTGTTCT			
GRIP28	NM_001281212.1	Forward	GCAGTTGGCTCACACCGCTTTG	125	62.4	2.05
		Reverse	AACAGTCCTGAACCGCCTCCAA			
F3′H	NM_001280987.1	Forward	AGGAGGAGGTTGCGGTGCTAAC	132	62.3	1.98
		Reverse	CGCCGAACACTCTCCTGCCTAA			
F3′5′H	NM_001281235.1	Forward	TCCATCGCATGGCTGGACATCC	101	62.55	1.93
		Reverse	GCCGTGTGCTCCTCCATCATCT			
UFGT	AF000372.1	Forward	TCAGGCGGAGGTCCTAGCACAT	81	62.3	2.11
		Reverse	GCCACGCTTTCCCACAATGAGT			
ACT	XM_002282480.4	Forward	GGCTGGATTTGCGGGTGATGAT	80	61.5	1.97
		Reverse	CCATGACACCAGTGTGCCTTGG			
AIG1	XM_002281960.4	Forward	GCACGGCTGAAGGCAGAAGAGA	104	62.4	1.99
		Reverse	TCCGTCTCCCTCTGTGCTCTCT			
GADPH	XM_002263109.3	Forward	AGGCTGGAGAAGGCTGCTACCT	139	62.4	1.89
		Reverse	TGCTGGACCTGTTGTCACCGAT			

## Data Availability

The data is contained within the manuscript.
